# Hybrid genome de novo assembly with methylome analysis of the anaerobic thermophilic subsurface bacterium *Thermanaerosceptrum fracticalcis* strain DRI-13^T^

**DOI:** 10.1186/s12864-021-07535-z

**Published:** 2021-03-23

**Authors:** Trevor R. Murphy, Rui Xiao, Scott D. Hamilton-Brehm

**Affiliations:** Department of Microbiology, Southern Illinois University Carbondale, Carbondale, IL USA

**Keywords:** Subsurface, Closed genome, Methylome, Terrestrial subsurface, BREX

## Abstract

**Background:**

There is a dearth of sequenced and closed microbial genomes from environments that exceed > 500 m below level terrestrial surface. Coupled with even fewer cultured isolates, study and understanding of how life endures in the extreme oligotrophic subsurface environments is greatly hindered. Using a de novo hybrid assembly of Illumina and Oxford Nanopore sequences we produced a circular genome with corresponding methylome profile of the recently characterized thermophilic, anaerobic, and fumarate-respiring subsurface bacterium, *Thermanaerosceptrum fracticalcis,* strain DRI-13^T^ to understand how this microorganism survives the deep subsurface.

**Results:**

The hybrid assembly produced a single circular genome of 3.8 Mb in length with an overall GC content of 45%. Out of the total 4022 annotated genes, 3884 are protein coding, 87 are RNA encoding genes, and the remaining 51 genes were associated with regulatory features of the genome including riboswitches and T-box leader sequences. Approximately 24% of the protein coding genes were hypothetical. Analysis of strain DRI-13^T^ genome revealed: 1) energy conservation by bifurcation hydrogenase when growing on fumarate, 2) four novel bacterial prophages, 3) methylation profile including 76.4% N6-methyladenine and 3.81% 5-methylcytosine corresponding to novel DNA methyltransferase motifs. As well a cluster of 45 genes of unknown protein families that have enriched DNA mCpG proximal to the transcription start sites, and 4) discovery of a putative core of bacteriophage exclusion (BREX) genes surrounded by hypothetical proteins, with predicted functions as helicases, nucleases, and exonucleases.

**Conclusions:**

The de novo hybrid assembly of strain DRI-13^T^ genome has provided a more contiguous and accurate view of the subsurface bacterium *T. fracticalcis,* strain DRI-13^T^. This genome analysis reveals a physiological focus supporting syntrophy, non-homologous double stranded DNA repair, mobility/adherence/chemotaxis, unique methylome profile/recognized motifs, and a BREX defense system. The key to microbial subsurface survival may not rest on genetic diversity, but rather through specific syntrophy niches and novel methylation strategies.

**Supplementary Information:**

The online version contains supplementary material available at 10.1186/s12864-021-07535-z.

## Background

The recently characterized thermophilic, anaerobic, fumarate-respiring bacterium, *Thermanaerosceptrum fracticalcis,* strain DRI-13^T^ was isolated from a ~ 876 m deep borehole intersecting the Death Valley Regional Flow system in the United States Nevada National Security Site [1]. Exploration and study of the subsurface has yielded many intriguing discoveries in ecology, geochemistry, energetics of microbial metabolism, and the origins of life [2–5]. Previous studies predict that subsurface life is primarily limited by the availability of energy and nutrients from the environment [6]. Despite these challenges, the subsurface supports a diverse microbial community spanning all three domains of life, but primarily consisting of *Bacteria* and *Archaea* [7, 8]. Across geochemical and spatial distances (cm to km), microbial diversities have been observed to change significantly [9, 10]. Microbial biochemical reactions drive complex nutrient recycling mechanisms and metabolic byproduct cross-feeding to sustain subsurface life in these oligotrophic environments [11–13]. However, extensive commensal relationships do not adequately explain a subsurface ecosystem steady state lasting thousand if not millions of years [14]. This mystery is further deepened when considering how microorganisms must manage energy expenditures to survive, defend against viral predation, DNA repair, avert entropy, and compete with other microorganisms for resources [15].

There are approximately 26,000 non-redundant bacterial genomes publicly available in the JGI/IMG database and only 19 of these contained metadata related to subsurface environments [16, 17]. Of these 19 genomes, the methylomes were not available. Sequencing technologies such as Oxford Nanopore technology (ONT) single-molecule real-time (SMRT), whole genome bisulfite sequencing (WGBS), or NEBNext Enzymatic Methyl-seq platforms have made available mapping three of the dominant features in prokaryotic methylomes [18–20]. No one method is the perfect tool, biases towards low CG content, over representation of methylation fraction, precision, organism of study, and indels can be found to varying degrees within each platform [21–26]. The three typically studied genomic methylation forms are N6-methyladenine (6 mA), N4-methylcytosine (4mC), and 5-methylcytosine (5mC) [27, 28]. With 6 mA being the major bacterial methylation target, it is associated with the 5′-GATC-3′ recognition sequences for DNA adenine methyltransferase (Dam). This is of interest because of 6 mA association with physiological processes, including bacteriophage resistance, mismatch repair, transposition, motility, and antibiotic resistance to name a few [29, 30]. Methylation at 5mC is typically associated 5′-CCWGG-3′ recognition sites for DNA cytosine methyltransferase (Dcm). Physiological processes of 5mC and 4mC have been elusive with some association with phage recombination, transposition, and protection against parasitism [31–33]. Historically, DNA methylation in bacteria is associated with restriction-digestion systems [34, 35]. Recent studies have also shown that bacteria utilize DNA methylation to regulate gene expression (e.g., cell length growth, biofilm formation, and host colonization) [36].

Efforts to expand the representation of deep subsurface microorganisms has been slow and synchronization between cultured isolates with circularized sequenced genomes has not always been present to offer strong inference. Given limited opportunities for obtaining pristine subsurface samples and the less-than-assured chances of isolating novel microorganisms, it is important to thoroughly use analytic tools and techniques to make each available microorganism a data point. Through each strategically characterized subsurface microorganism we will have a better understanding of how life survives under the extreme physiological pressures of Earth’s subsurface [37, 38]. Combining physiological, genomic, and metabolic characterization strategies from cultured isolates establishes a comprehensive baseline that allows unrestricted future research opportunities [39]. Microbial community sequencing alone has limitations resolving molecular mechanisms of functionality, which is complicated by novel genetic, metabolic, and sampling bias strategies [40–42]. By contrast, the study of cultivated microbial isolates from Earth’s surface has extended our understanding of life’s mechanisms and origins, yet the ‘great plate count anomaly’ shows that considerably more could be learned [43–45]. The same problems in culturing novel surface microorganisms applies to subsurface microorganisms as well, if not more so. Genomes serve as a platform from which specific hypotheses can test physiological processes of microorganisms within their ecosystem [46]. Furthermore, a thorough understanding of a microorganism is achieved by ‘closing’ the genome, making available intergenic non-coding and synteny information.

Here we describe the de novo hybrid assembly of the recently characterized subsurface bacterium *T. fracticalcis* strain DRI-13^T^*.* Strain DRI-13^T^ is a novel genus in the *Peptococcaceae* family with an average nucleotide identity of 66% with its two closest relatives (*Pelotomaculum propionicicum* GCA_004369225.1 and *Pelotomaculum thermopropionicum* GCA_000010565.1). A combination of Illumina and Oxford Nanopore sequencing technologies were used to generate a circularized assembly including methylome profile. Analysis of *T. fracticalcis* genome has revealed four imbedded novel prophage artifacts, unique methylation biases, and potential metabolic approaches to managing energy conservation in the subsurface.

## Results

### Genome sequencing

The revisited and reprocessed Illumina sequencing data collected from a previous strain DRI-13^T^ genome assemblage consisted of 45 million 150 bp sequences that provided an estimated 1858X coverage based. Analysis with FastQC software indicated that the Illumina sequences did not contain any ambiguous bases and were free of adapter sequences. The MinKNOW software reported 1525 open pores resulting in 1.12 GB of FASTQ data after 3 hours of Oxford Nanopore sequencing. The raw data consisted of 202,000 reads with an average length of 1.7 kb (the longest read consisting of 45 kb), for a total of 5.3 Mb and an estimated 94X genome coverage of DRI-13^T^.

### Genome assembly

Multiple assemblies of the Nanopore and Illumina sequences were analyzed for contiguity, gene count, and alignment agreement with the prior assembly to reach a final genome consensus (Table 1). These measurements were generated using Quast prior to selecting an assembly for annotation. Short read SPAdes assembly was discarded because of its highly fragmented nature, producing over 2000 contigs. SPAdes-Hybrid also resulted in a fragmented assembly with high numbers of contigs but was able to produce a large contig of the expected length; however, it contained gaps filled with ambiguous bases, which could not be resolved. MaSuRCA generated a less fragmented assembly than SPAdes-Hybrid, but still contained unresolved regions and contained 2000 mismatches and indels compared to the Unicycler assembly used for final annotation. The Canu assembly contained nearly 400 more genes relative to the other assemblies; most were determined to be less than 300 bp in length with indels, which were fragmenting genes. Flye and Unicycler each generated contiguous assemblies of similar size and gene count. The Flye assembly contained nearly 100 indels and mismatches along with a poor alignment profile with the Unicycler assembly. Previous studies outlining Unicycler’s assembly capabilities confirmed the selection of the final hybrid assembly for this study [47, 48]. The predicted Gene Counts by Quast’s gene estimation algorithm is only an estimation and does not reflect nor affect the final official JGI annotation gene total. DRI-13^T^ genome is available from JGI 2842667859 and NCBI GCA_000746025.2.

**Table 1:** *T. fracticalcis* DRI-13^T^ Hybrid genome assembly comparison

Assembler	Assembly Size	Contigs	Largest Contig Length	GC Percentage	Ambiguous Base Count	Predicted Gene Count
Unicycler^a^	3,805,411	1	3,805,411	45.2%	0	2216
Flye	3,762,930	1	3,762,930	45.2%	0	2230
Canu	3,746,854	1	3,746,854	45.3%	0	2610
MaSuRCA	3,734,429	5	3,730,424	45.2%	100	2247
Spades-hybrid	3,917,015	171	3,697,741	45.0%	24,644	2254
SPAdes Illumina only	5,294,664	2060	192,791	42.2%	110	2933
Scaffold genome assembly^b^	3,649,665	105	193,268	45.1%	265	2209

^a^This work

^b^Information acquired from Hamilton-Brehm et al. 2019 [1]

### Genome structure

**Fig. 1:**
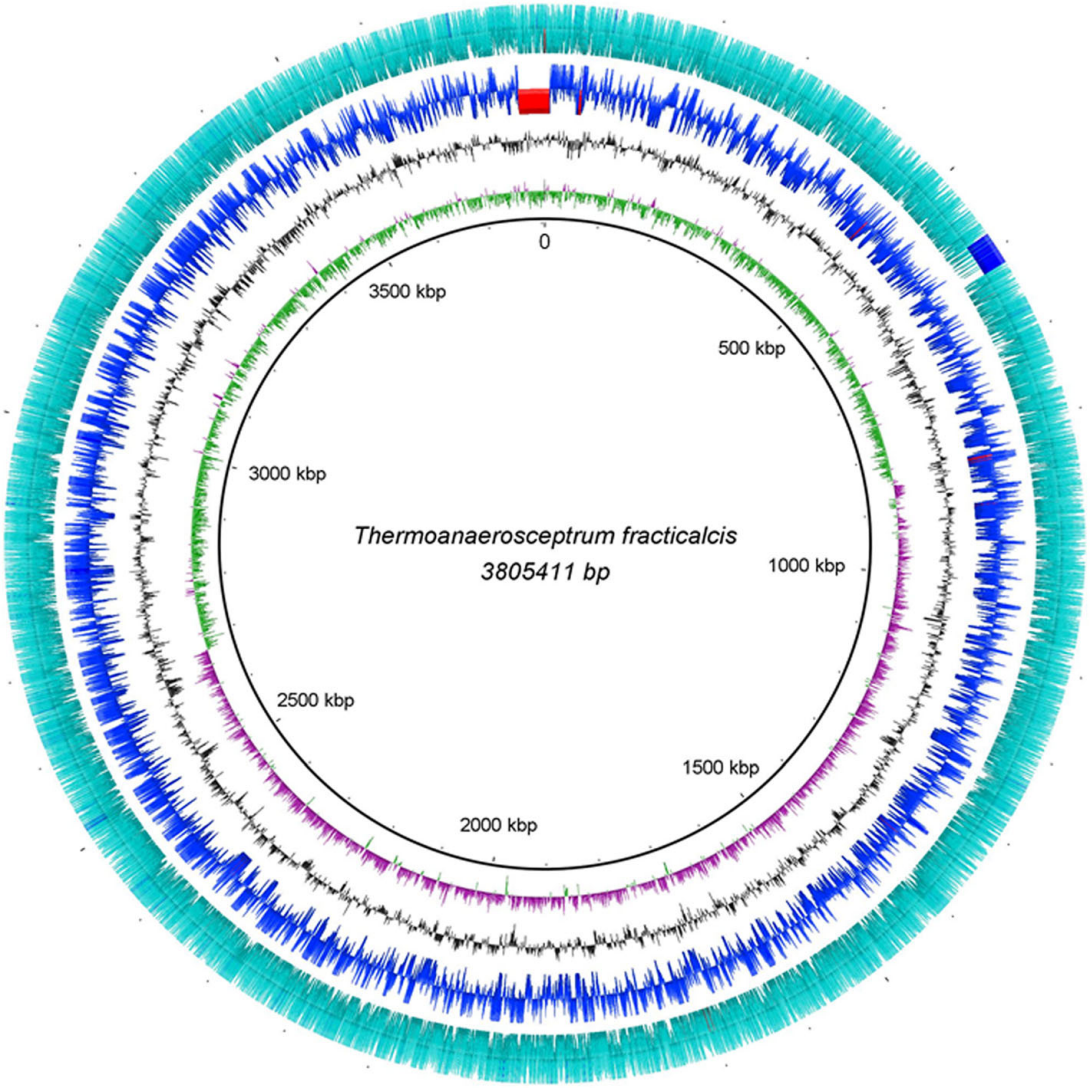
Graphical representation of hybrid chromosome assembly. Starting from the inside to outside, the first ring (violet and green) shows the GC skew of the genome assembly, second ring (black) representing GC content, third (blue) displays Illumina coverage and fourth rings (aqua) displays the Nanopore coverage depth. Solid red and blue bars indicate areas of differential coverage depth. Genome orientation position is marked as ‘0’

The Unicycler genome assembly produced a single circular contig measuring 3.8 Mb (Fig. 1). The start site (0 position) as selected by Unicycler was a hypothetical protein with no homology with other known proteins. Annotation by the Joint Genome Institute (JGI) revealed 4022 genes; of these, 3884 are protein encoding (927 hypothetical proteins), 87 RNA encoding genes, and 51 regulatory features (riboswitches and T-box leader sequences), with a GC content of 45% for the entire genome (Table 2).

**Table 2:** Genome metrics of *T. fracticalcis* DRI-13^T^

Feature	Hybrid genome 2020 assembly^**a**^		Scaffold genome 2014 assembly^**b**^	
	Value	% of Total	Value	% of Total
Contigs	1	–	105	–
Genome size (bp)	3,805,411	–	3,649,665	–
G + C %	45.0%	–	45.1%	–
Total number of genes	4022	100%	3749	100%
Protein coding genes	3884	97%	3671	98%
Protein coding gene with predicted function	2957	74%	2876	77%
Protein coding gene without predicted function	927	23%	795	21%
RNA genes	87	2%	78	2%
Mobile elements	78	2%	33	1%
CRISPR arrays	2	–	1	–
Prophages	4	–	4	–

^a^This work

^b^Information acquired from Hamilton-Brehm et al. 2019 [1]

### Functional gene annotation

**Fig. 2:**
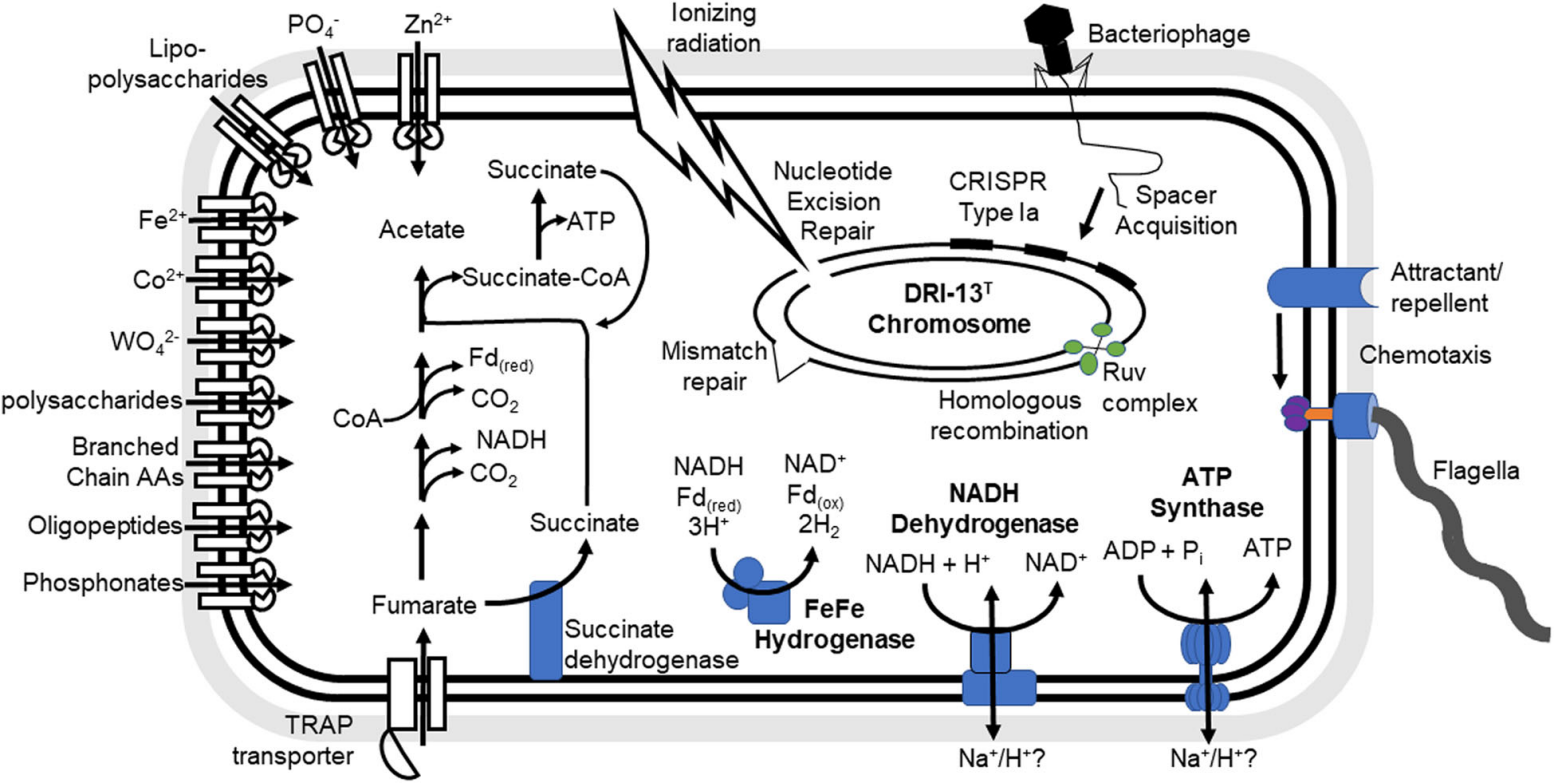
Diagram of genome mechanisms. Genomic potential outlining hypothesized cellular functions contributing to *T. fracticalcis*’s survival in the subsurface. Outlined functionalities include fumarate metabolism, DNA replication and repair, and cellular import and export. Gene ID and accession numbers for included proteins can be found in Supplemental Table S1

Cell survivability and metabolism-annotated genes including carbohydrate and peptide transportation, phosphate and nitrogen transport, DNA repair and recombination, chemotaxis, electron transport/ATP synthesis, clustered regularly interspaced short palindromic repeats (CRISPR) Cas proteins and arrays, Bacteriophage Exclusion (BREX) system, and prophages were selected for analysis (Fig. 2). The annotation and JGI gene number for genes represented in the cell diagram and prophages can be found in Supplemental Table S1.

Key catabolism/anabolism, hydrogenases, and respiratory genes were analyzed revealing that all genes necessary to carry out glycolysis were present, although the gene encoding Phosphoenolpyruvate (PEP) carboxykinase, the enzyme that catalyzes an irreversible step in gluconeogenesis to form PEP from oxaloacetate, was missing. The amino acid sequence of PEP carboxykinase from *E. coli* J96 (NCBI genome accession number: GCA_000295775.2, Locus: ELL41697) was compared to strain DRI-13^T^ by BLAST checking for mis-annotation, but yielded no result. Respiratory complexes I and II shared between 48 and 74% and 61–74% amino acid sequence identity (AASI), respectively, with those from other bacteria in the class Clostridia, notably *Calderihabitans maritimus* (GCA_002207765.1, Locus: WP_088554119) and *Desulfonispora thiosulfatigenes* (GCA_900176035.1, Locus: WP_084053933). No cytochromes were annotated in the genome other than cytochrome bd complex. Two gene clusters encoding bifurcating hydrogenases in the DRI-13^T^ genome having a range of 38–66% AASI to *Thermotoga maritima* (GCA_000230655.3,) and members of the genus *Caldicellulosiruptor*.

When exploring DNA repair mechanisms, the DRI-13^T^ genome encodes for DNA primase, ligase, and DNA polymerases I and III required to initiate and complete DNA replication, however, there are no genes encoding DNA pol III holoenzyme’s proofreading capability. For single, stranded DNA damage, the genome encodes DNA ligase, DNA pol-I, DNA 3-methyladenine glycosylase I, TDG/mug DNA glycosylase, Deoxyribonuclease-4, Endonuclease-3, Formamidopyrimidine-DNA glycosylase, and Single-stranded-DNA specific exonuclease required for both long and short patch base excision repair. All necessary genes for the UvrA-D proteins involved in nucleotide excision repair pathway were present. Strain DRI-13^T^ possesses genes for mismatch repair but it is notably missing the gene for *mutH*; however, the endonuclease function may be made up for in *mutL* gene product. BLAST analysis of DNA mismatch repair gene amino sequences were similar to other related anaerobic thermophiles 43–60% AASI for *Calderihabitans maritimus* (GCA_002207765.1), and 45–88% AASI for both *Thermincola ferriacetica* (GCA_001263415.1), and *Thermincola potens* (GCA_000092945.1). Genome comparisons with close relatives determined that all the aforementioned DNA repair pathways are conserved.

Chemotaxis and transport was investigated, revealing that response to an attractant or repellent is carried out by the Che two-component system. Amino acid sequences of the *T. fracticalcis* Che system shared sequence identities ranging from 41 to 84% with bacteria within the class Thermoanaerobacterales and Clostridiales. All close relatives possessed the Che two-component system. A type IV pilus bracketed by identical transposons, may have been acquired through a lateral gene transfer event. BLAST results of pilus translated amino acid sequences from the genes *pilB* and *pilT* found with 55–65% AASI to *Calderhabitans maritimus* (GCA_002207765.1, Locus: GAW94128) but four genes (*pilC*, *pilX*, *pilM*, and *pilN*) translated amino acid sequences lacked results with more than 30% AASI. Strain DRI-13^T^ encodes only *tatA* and *tatC* genes and thus contains only a minimalistic Tat pathway. Also, the Sec pathway is missing *secE* but contains *secD* with 60% AASI to *Thermincola potens* (GCA_000092945.1,Locus: WP_013120086) and *secF* with 65% AASI to *Calderhabitans maritimus* (GCA_002207765.1, Locus: WP_088553991). The *T. fracticalcis* genome encodes for several ATP-binding cassette transporters (ABC transporters) that import necessary nutrients such as iron, zinc, polysaccharides, lipopolysaccharides (LPS), oligopeptide ABC transporters, and phosphate. Genes for ammonia transport proteins were found in bacteria within the order Clostridiales (52–80% AASI) and Thermoanaerobacterale*s* (59–80% AASI) and representatives of the class Negativicutes (62–80% AASI). Ten genes for nitrogenase function and maturation were present in the genome, sharing 65–90% AASI with bacterial Firmicutes *Sporomosa acidovorans* (GCA_002257695.1, Locus: WP_093792115, *Methylomusa anaerophila* (GCA_003966895.1,Locus: BBB92032) and members of the *Desulfotomaculum* genus. Nitrogenase genes were present in all of the genomes used for DRI-13^T^ comparisons.

### Cellular defenses and prophages

Two CRISPR arrays are present in the genome, as determined by both JGI and CRISPRCasFinder tool using CRISPR-Cas++. The first array (genome location: 638,577 bp - 639,957 bp) is 1.4 kb in length containing 18 spacers and 19 repeats (DNA sequence repeat 5′-GTTGCAATGCCTAGCTCAGAGGTTTAAAGACTGAGAC-3′). The second array (genome location (1,393,508 bp - 1,399,131 bp) is 5.6 kb with 75 spacer sequences and 76 repeats (DNA sequence repeat 5′-CTTTCAGTCCCCATGTATCGGGTCTATTCAATGGAAC-3′). Each array has a distinct repeat sequence. Upstream by 250 bp of the second array is a series of Cas genes, including *cas1*, *cas2*, *cas3*, *cas4*, *cas6*, and *cas7*. CRISPR spacers were unsuccessfully mapped to putative exogenous DNA elements and anywhere else in the genome aside from their position in the array. This result indicates that the spacers in the CRISPR array do not match the any of the prophages present in the genome. Including, an absence of self-targeting spacers sequences mapping outside of the CRISPR array. BLAST searches of spacer sequences found matches for 14 of the 93 total spacers. Results of this analysis did not find that any of the matching spacers came from organisms that would exist in a similar environment as DRI-13^T^. In addition to a CRISPR system a putative Bacteriophage Exclusion (BREX) system was identified in strain DRI-13^T^ genome. Three putative BREX genes (*brxHI*, *brxD*, *pglX*) in a 36kbp gene cluster match’s core annotated BREX system descriptions. Within the cluster several hypothetical proteins with predicted functions as helicases, nucleases, and exonucleases also support identification of a BREX system.

**Fig. 3:**
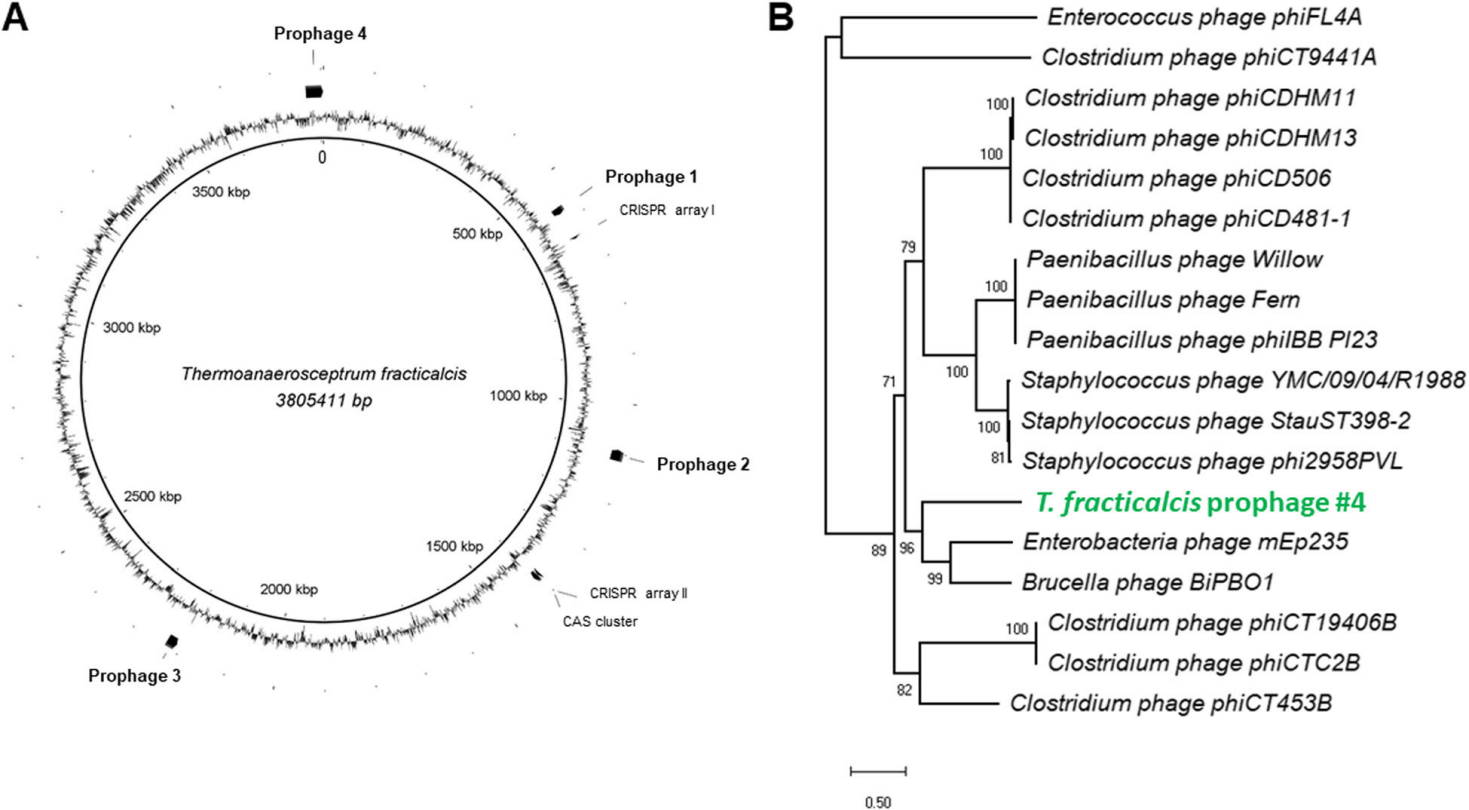
Viral prophage positions and terminase alignments. **a** Genome locations of CRISPR-Cas and putative prophages identified with Phaster. **b** Large subunit terminase amino acid sequence from intact DRI-13^T^ prophage #4 compared to bacteriophages with at least two similar protein sequences identified by Phaster. Phylogenetic analyses were aligned by ClustW, unrooted maximum likelihood phylogenetic tree by MEGAX, with 1000 bootstraps

Four prophage elements were identified by PHASTER web tool. Three of these prophages (prophage #1 starts at position 566,157 bp, prophage #2 starts at position 1,114,566 bp reverse direction, and prophage #3 starts at position 2,229,417 bp reverse direction) were incomplete. The fourth prophage is intact (prophage #4 starts at position 3,768,183 bp). Prophage #1 is 14.9 kb in length with a GC content of 43% and contains genes encoding proteins for DNA replication including topoisomerase (JGI ID 2842668476) and helicase (JGI ID: 2842668481). Prophage #2 is 22.3 kb with a GC content of 44% and contains hypothetical proteins, repressor proteins (JGI ID: 2842669072 and 2,842,669,073) and integrase proteins (JGI ID: 2842669078). Prophage #3 is 20.5 kb in length with 45% GC content and lacks genes for structural components but contains regulatory genes for repressing prophage induction (JGI ID: 2842670225). Prophage #4 is identified by PHASTER to be an intact prophage at 36.1 kb in length with a GC content of 44% and contains genes for encoding capsid proteins (JGI ID: 284267182) and tail fibers (JGI ID: 2842671872) along with transcriptional control elements (JGI ID: 2842671829 and 2,842,671,828) and genes for cell wall hydrolases (JGI ID: 2842671873 and 2,842,671,874). Prophage element positions within the genome are noted in Fig. 3a. A maximum likelihood phylogenetic tree was constructed based on amino acid sequence of the large subunit of the terminase protein from bacteriophages that contained at least two similar amino acid sequences from prophage #4 which resulted in 32% AASI to Enterobacteria phage mEp235 (GCA_000903595.1) (Fig. 3b).

### Methylome profile

**Fig. 4:**
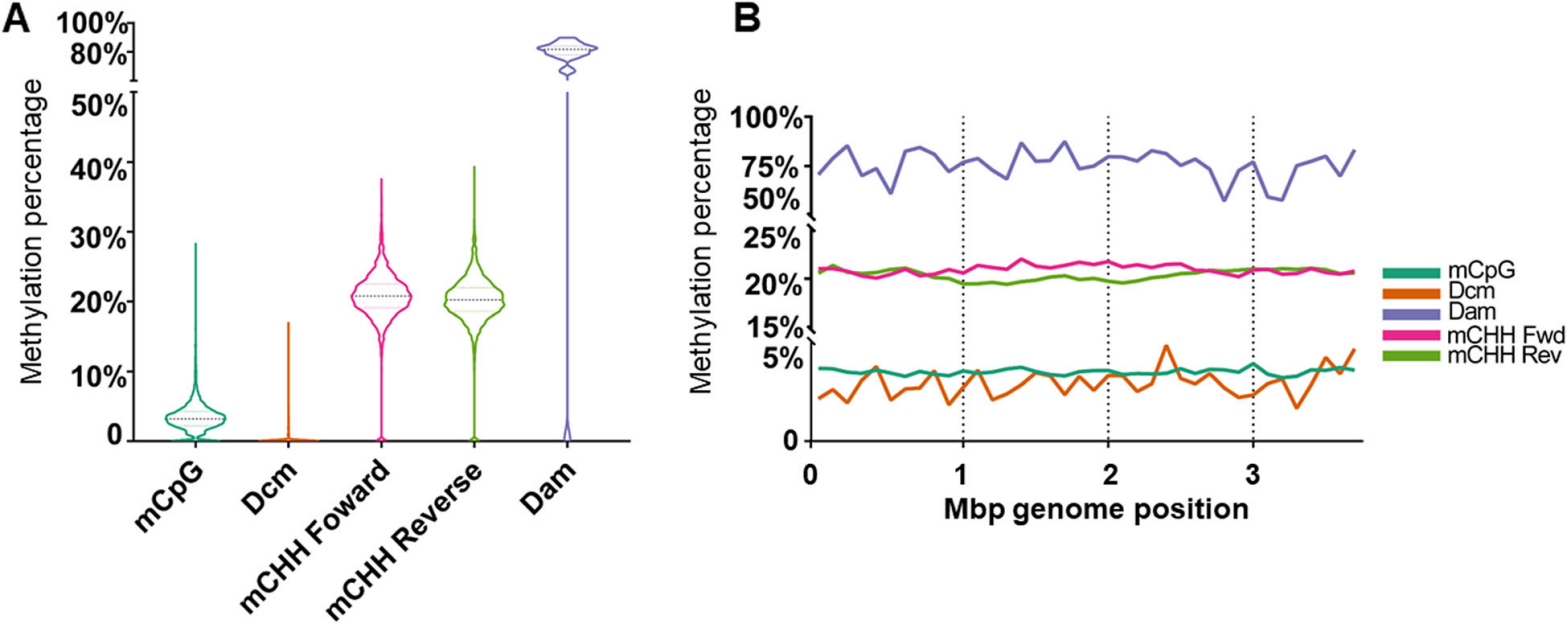
DNA methylation context distribution and whole genome plot of strain DRI-13^T^. **a** Methylation level distribution for strain DRI-13^T^. Black dash line indicates median value. Upper and lower colored dash line indicates 1st and 3rd quartile. **b** Strain DRI-13^T^’s genome is partitioned into 100kbp non-overlapping bins. DNA methylation level for each context is summarized and plotted over the entire genome

Strain DRI-13^T^ methylation distribution plot showed that the microorganism’s methylation level varies between different contexts (Fig. 4a). Symmetrical DNA methylation was observed where C or A on both strands at the palindromic motif are methylated. Meanwhile asymmetrical DNA methylation only has half of the strands methylated. Results show lower average symmetrical 5mC (mCpG) compared to asymmetrical 5mC (mCHH forward, mCHH reverse). Although the total amount of predicted methylated Dam sites (5′-GATC-3′) identified are low (Table 3), the average methylation percentage for Dam is higher. No clear patterns were found for Dcm and Dam context across all genes (Supplemental Figure S1). Additional modified motifs, besides canonical DNA methylation in mCpG, Dcm, mCHH, and Dam context using de novo modified base detection have identified two significant enriched motifs G (mA) ACT and C (mC) GG (Supplemental Figure S2).

**Table 3:** Mapped methylation motifs and percentages

Mapped Motif	Number of Motifs	Methylation Percentage	Context Specific Coverage
CpG	133,910	4.28%	121X
Dcm	18,831	3.81%	94X
Dam	4903	76.40%	42X
CHH Fwd	363,179	17.41%	54X
CHH Rev	365,658	17.39%	53.5X

The two putative DNA methyltransferases (Dam and Dcm) were identified based upon genome annotation using NCBI Constraint-based Multiple Alignment Tool (COBALT). The core protein domain for methyltransferase activity was highly conserved compared to the methyltransferases of other prokaryotes and eukaryotes (Supplemental Figure S3).

**Fig. 5:**
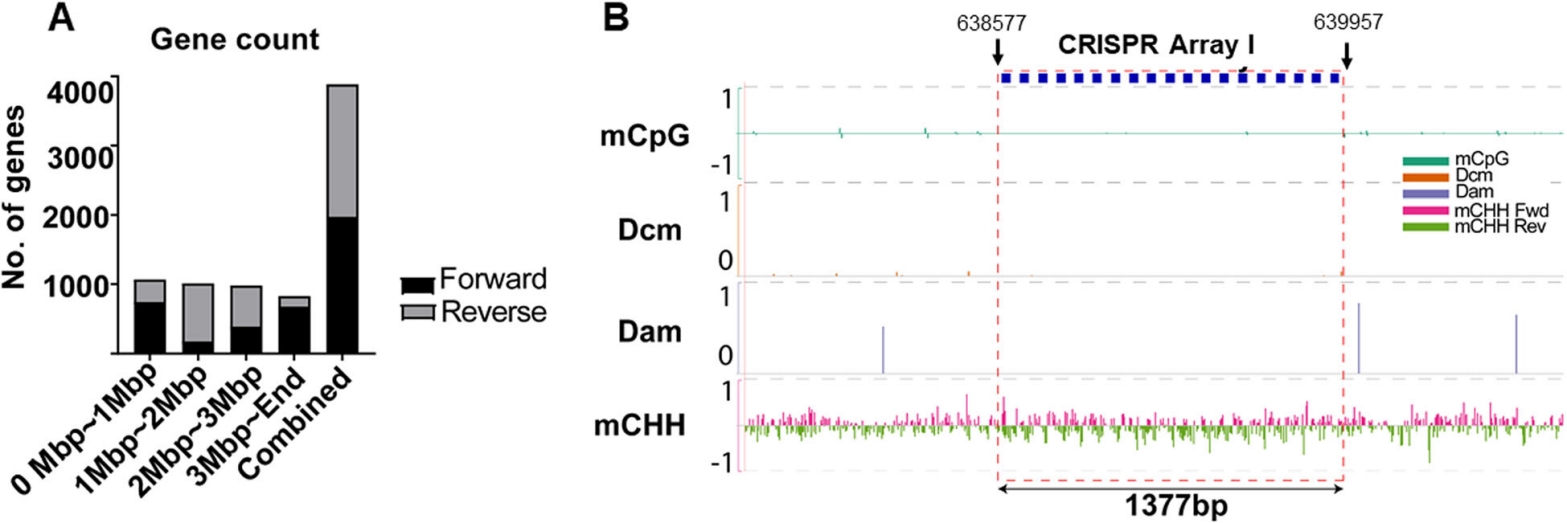
Gene count distribution and CRISPR array methylation profile. **a** Gene counts distribution based on specific strands of strain DRI-13^T^ in 1Mbp non-overlapping pins. Last column shows total amount of genes on forward and reverse strand. **b** Methylation profile of one of strain DRI-13^T^’s CRISPR array. Blue box are CRISPR spacers. Empty space between blue boxes are CRISPR repeats. Row (1) shows 100% methylation on positive strand while − 1 shows 100% methylation on reverse strand. Row (0) shows no methylation. Red dashed lines show the boundary of the CRISPR array

Plotted methylation signals in 100 k bp bins across DRI-13^T^’s genome revealed mCpG and Dcm levels were below 0.5% across genome, while Dam levels are around 75%, and mCHH on the reverse strand are depleted (Fig. 4a). The number of annotated genes in 1 million bp bins across all 3 Mbp of the DRI-13^T^ genome for both strands show an unequal distribution (Table 4 and Fig. 5a). On the 1–2 Mbp bin, 836 genes are located on the reverse strand while only 175 genes are present on the forward strand; the large proportion of genes present on the reverse strand at this bracket negatively correlates with the mCHH level observed in chromosomal methylation plotting (Fig. 4b).

**Table 4:** Number of genes on forward and reverse strands in 1Mbp segments

Genome position	Forward	Reverse	Sum	Difference
0 - 1Mbp	740	325	1065	415
1 - 2Mbp	175	836	1011	661
2 - 3Mbp	385	596	981	211
3–3.8Mpd	675	152	827	523
Total	1975	1909	3884	66

DNA methylation for mCpG, Dcm and Dam is depleted at CRISPR regions. Integrative Genomics Viewer screenshots analyzed CRISPR array 1 (genome location: 638,577 bp) in DRI-13^T^. Interestingly, CRISPR spacers were methylated for both strands with mCHH, while the repeat sequence between spacers remained unmethylated (Fig. 5b). The methylation signal profile for the annotated genes using methylation metaplot of the upstream and downstream 1000 bp showed a pattern of mCpG and mCHH depletion near both promoter regions and transcription end sites. A depletion of DNA methylation of all context was observed near transcription start sites as well as transcription termination sites (TSS). In contrast, there is a small cluster of 45 genes, named ‘cluster 1’, that have enriched DNA mCpG proximal toward TSS (Supplemental Figure S4). Application of InterProScan and Gene Ontology (GO) on cluster 1 genes shows most of the genes in cluster 1 encode families of unknown proteins (Supplemental Table S2).

## Discussion

Despite increasing interest of subsurface ecology and microorganisms, there is a paucity of physiological and genomic characterizations of subsurface microorganisms. For example, an exciting newly discovered member to the genus *Thermoanaerosceptrum* was isolated from a deep subsurface environment located in Republic of Buryatia, Russia with seemingly different metabolic capabilities, the genome was not sequenced [49]. By generating a circularized genome and corresponding methylome of DRI-13^T^, we sought to provide one complete view of an interesting subsurface microorganism. To do this we had to select an algorithm that produces the most accurate de novo hybrid assembly from Oxford Nanopore and Illumina NGS assembly (Table 1). The final size of the hybrid genome was 3.8 Mbp as compared to 3.6 Mpb from the previous 105 contig Illumina assembly. This resulted in an increase total number of annotated genes from 3749 to 4022 (Table 2). The previously missing 273 genes had annotated functions associated with cell division, membrane transport and DNA repair. There were also gene annotation discrepancies from the first assembly in 2014 compared to the hybrid assembly in this 2020 study. Overall, it is interesting to note that while the first Illumina sequencing assembly had a predicted 1858x coverage of the genome, genetic information was still missing and was covered through a combination of long and short sequencing methods.

Strain DRI-13^T^ was characterized to utilize fumarate as the major (if not only) carbon and energy source. This seems counter intuitive since living in the oligotrophic subsurface environment would likely promote opportunistic strategies of any carbon or energy source and that fumarate was not found in appreciable concentrations in the environment. Agreeing with the prior genome assembly, all required genes for fumarate respiration were present in the genome, consistent with laboratory culturing observations. A subset of genes thought to support survival in oligotrophic subsurface were investigated and outlined in Fig. 2. All genes necessary to carry out glycolysis via Embden-Meyerhof pathway were found to be present, although strain DRI-13^T^ showed only limited growth in the presence of glucose. Annotated ABC transporters for ribose, sn-glycerol-3-phosphate, polysaccharide, lipopolysaccharide, and fructose import via phosphotransferase systems were the only annotated carbohydrate transport genes identified. None of which when appear to stimulate growth of strain DRI-13^T^. No glucose specific transporter were annotated. If absent this would suggest glucose enters the cell through transporter(s) with relaxed substrate specificity or via novel proteins capable of glucose transport. Potentially, sugars can be generated through gluconeogenesis, though Phosphoenolpyruvate (PEP) carboxykinase which catalyzes the irreversible step in the formation of PEP from oxaloacetate is missing. A gene present in the genome for Pyruvate orthophosphate dikinase, which has been shown to catalyze the formation of PEP in other organisms, may allow for gluconeogenesis pathway to function in strain DRI-13^T^ [50, 51]. Additionally, the gene encoding the lactate dehydrogenase enzyme is not present, supporting the findings in the characterization study that lactate production does not occur [1]. Kegg and MetaCyc pathways suggest that strain DRI-13^T^ does not have a functional citric acid cycle (TCA) based on the inability to synthesize oxaloacetate from malate, and lack of citrate synthase to condense oxaloacetate and acetyl-coA to form citrate. Culture experiments using malate + glucose and oxaloacetate + glucose showed that only the later stimulated growth (data not shown). No genes for citrate synthase or 2-methylcitrate synthase were detected in the genome [52]. This should lead to an inability for acetyl-coA to enter the TCA cycle, thus restricting the usefulness of these biochemical reactions in strain DRI-13^T^. While oxaloacetate has multiple cell fates that may account for the improved growth, if it is found that it enters through the TCA cycle then it would be evident that a novel ortholog for citrate synthase is present.

Close relatives of DRI-13^T^ *P. propionicicum* and *P. thermopropionicum* (representing surface analogues) and *Desulfotomaculum reducens* and *Thermincola ferriacetica* (representing subsurface analogues) were used in genomic comparisons to better elucidate metabolic architecture. In all cases, homologous genes used in gluconeogenesis, and TCA cycle were present. Each of these pathways, aside from the TCA cycle, were predicted to be functional based on KEGG pathways. Within the TCA cycle, each organism encoded malate dehydrogenase except for *T. ferriacetica* that does not encode malate dehydrogenase but possesses citrate synthase, as compared to DRI-13^T^ which is missing both genes encoding for these enzymes. Interestingly, both *Pelotomaculum* species and *T. ferriacetica*, under certain conditions, are syntrophic organisms [53]. Given that the same missing components that would complete metabolic pathways and close phylogenetic relatives engage in syntrophic relationships, it is reasonable to suspect that DRI-13^T^ also uses syntrophic activities to survive subsurface environments.

Strain DRI-13^T^ has multiple methods to oxidize electron carriers and remove electrons via H_2_ production. A proton/sodium motive force can be maintained by complex I NADH dehydrogenase and complex II donating electrons to fumarate forming succinate. It is noted that genes for complexes III and IV could not be identified in the genome. The presence of Fe/Fe hydrogenase subunits α, β, and γ homologs to equivalents in *Thermotoga maritima* is interesting as it suggests that when fermenting fumarate, strain DRI-13^T^ oxidizes electron carriers via bifurcation, ultimately producing H_2_ [36, 54, 55]. Hydrogen production has been observed in cultures of DRI-13^T^ when utilizing fumarate. Reliance on other microorganisms to supply needed intermediates for metabolic functionality, in return for production of H_2_ would be an important product in subsurface niches, potentially assisting in reciprocal production of fumarate by other anaerobic microorganisms to sustain DRI-13^T^ [56]. Homologs of the bifurcating hydrogenase was also present in all close relatives.

Flagellar motility in DRI-13^T^ has been observed previously. This study revealed a complete flagellar assembly and the Che two component system. No genes for the histidine kinase specific for aerotaxis was present in the genome. If aerotaxis is not conducted, it remains unclear if DRI -13^T^ responds to chemical attractants or repellents. A type IV pilus was annotated in DRI-13^T^, but no twitching motility nor biofilm formation has been observed. Each of the close relatives used for genomic comparisons contained flagellar motility genes, however only *T. ferriacetica*, found in hot springs, contained a type IV pilus and has been observed to form biofilms [57]. Comparatively, the pilus in DRI-13^T^ was between two identical transposon sequences, and this was not the case for *T. ferriacetica*. As such, this suggests that DRI-13^T^ may have acquired its pilus by horizontal gene transfer. While biofilms come at an energetic cost, they have been found in the subsurface with greater cell density, diversity and in fractured mineral ecosystems similar to where DRI-13^T^ was isolated [58, 59]. By encoding this pilus DRI-13^T^ gains the capacity to adhere to surfaces, increasing its chances of being part of a multi-species biofilm where odds of finding nutrients or participating in syntrophic interactions have increased probabilities by proximity.

One of the most intriguing hypotheses attributed to subsurface microorganisms is that cell division occurs over large time scales (thousands of years) because of the lack of surplus energy [14, 60]. A recent study of subseafloor microbes found that microbial life could persist in a metabolically active state for millions of years [61]. During these long residency periods, DNA repair would be necessary for maintenance of the genome. DRI-13^T^ and its close relatives contained the same DNA repair pathways but each could only repair double-stranded breaks by non-homologous end joining. This was indicated by the absence of genes *RecB* and *RecC*, and the presence of genes for *Ku* and *ligD*. This may indicate that double-stranded breaks in the microorganism’s genome, possibly caused from ionizing radiation, are repaired through non-homologous recombination [62].

Embedded within the DRI-13^T^ genome are four predicted prophages (Fig. 3a). However, three of the prophages are incomplete or the sets of phage structural component genes have eroded. The fourth prophage is intact with a 36.1 kb long genome, containing 51 genes, of which 20 are hypothetical proteins with no known homologs. Novel viral genes are characteristic of the undiscovered genetic potential hypothesized to exist in the subsurface [63]. Amino acid comparisons find Enterobacteria phage mEp235 (GCA_000903595.1) shares the greatest sequence similarity with the intact prophage (Fig. 3b). The impact of these prophages upon strain DRI-13^T^ fitness is unknown.

The inclusion of the Nanopore sequencing technology for completing the genome allows the acquisition of canonical direct base modification information [64]. To our knowledge this is the first time a deep (> 500 m) subsurface microorganism’s methylome has been analyzed. Strain DRI-13^T^’s genome contains multiple DNA methylation contexts, including low and consistent amounts of mCpG across the entire genome, and even lower levels of Dcm (Fig. 4). Low Dcm may be explained by the lack of Dcm specific DNA methyltransferase in the genome that methylates 5′-CCWGG-3′ at the second cytosine from the left. What was observed in terms of Dcm methylation may be contributed by DRI-13^T^’s own unique methylation motif similar to mCHG context. DRI-13^T^ shows a high level of Dam relative to the only few Dam sites found in the genome (Table 3). Upon investigation 6 mA were found in a motif of 5′-GAACT-3′ instead of the expected 5′-GATC-3′ (Supplemental Figure S2). Such high level of Dam may suggest adenine methylation plays a role in important cellular functions, such as regulating DNA replication, cell division, or defense against viral predation.

Unlike the other DNA methylation types that are present on both strands due to their motif’s palindromic nature, mCHH is asymmetrical [65]. Studies in eukaryotes have shown that mCHH is usually associated with selfish DNA suppression or adjustment of gene expression [66]. Strain DRI-13^T^ had a relatively high amount of mCHH on the forward strand at 1Mbp - 2Mbp segments, while on the reverse strand showed mCHH depletion (Fig. 4b). For strain DRI-13^T^ we see clear depletion of DNA methylation of all context near transcription start sites as well as transcription termination sites (TSS). Promoter sequences are essential in regulating gene expression, and the depletion of DNA methylation near strain DRI-13^T^ promoters also agrees with previous publications characterizing bacteria DNA methylation [67]. Even though much of strain DRI-13^T^’s protein encoding genes lack DNA methylation at TSS, there is a small cluster of 45 genes, named ‘cluster 1’, that have enriched DNA mCpG proximal toward TSS. The enriched mCpG at promoter regions of cluster 1 gene may function as cis-regulatory elements that either enhance or suppress their associated genes’ expression (Supplemental Figure S4). We applied InterProScan [68] on cluster 1 genes, and Gene Ontology (GO) enrichment shows most of the genes in cluster 1 encodes families of unknown proteins (Supplemental Table S2). Strain DRI-13^T^ may thus use DNA methylation to regulate gene expression by controlling promoter methylation level.

We analyzed DRI-13^T^’s CRISPR array spacer methylation contexts located at 600kbp and found them to be completely devoid of mCpG, Dcm, and Dam. As a crucial part of a bacterium’s adaptive immune system, CRISPR array cannot be altered or else defense against viruses/plasmids is lost. This explains the lack of methylated DNA for most contexts. On the other hand, DRI-13^T^’s CRISPR array showed periodicity of mCHH signal across the array. The context mCHH is usually deposited in association with RNA polymerase activity. It is possible that RNAPol traveling through DRI-13^T^’s CRISPR array generates repeat RNAs and causes mCHH methylation on adjacent CRISPR spacers [69]. To determine the possible source of these spacer sequences, they were mapped to all four putative prophage elements and compared by BLAST. No spacer reads mapped to any of the prophage elements in DRI-13^T^ or any other location in the genome aside from their positions in the CRISPR array. Of the 93 spacer sequences only 14 matched any other known spacers in the CRISPRdb database [70]. The bacteria sharing these spacers, were not associated with the subsurface. The repeat sequences present in each array are different from each other, yet only one set of CAS genes are present located near CRISPR array #2. It is not clear, why two repeats are present, unless the current CAS genes can utilize both arrays. It is also unclear if one array is active and the other is not. We have analyzed the methylation distribution of array #1 and array #2 signals in asymmetrical context (mCHH) which showed depletion at repeats, but significantly higher mC level in the spacers. This could mean that the same set of CAS genes are responsible for maintenance of both CRISPR arrays as mC signal distribution of the two arrays are similar. Further experimentation is needed to better understand this.

Recently another microbial self-defense mechanism, called Bacteriophage Exclusion (BREX) system has been identified [71]. Although the exact mechanism of action is not well understood, BREX is thought to methylate host DNA asymmetrically to distinguish host DNA from newly acquired phage integration [72]. The BREX system is usually composed of a core system consisting of proteins containing an ATPase domain, a DNA methyltransferase domain and phosphatase domain [71]. The system usually resides in 100kbp long immune system gene clusters which are also composed of many proteins without known function varying by phylogeny. While BREX does not appear to stop phage integration, it prevents phage activation viral-DNA [72]. DRI-13^T^ genome has three putative BREX genes (*brxHI*, *brxD*, *pglX*) in a 36kbp gene cluster that matches the core BREX system description (Supplemental Table S1). Hypothetical proteins, with predicted functions as helicases, nucleases, and exonucleases, surround these putative core BREX genes. The presence of a BREX system in DRI-13^T^’s genome may explain the observed integration of phage DNA in the genome. The BREX system may keep prophages dormant, allowing mutations to permanently remove phage functionality. In the context of the subsurface, where the tempo of evolution is proposed to be in the ‘slow lane’, this may provide a solution to fast track generation of ‘new’ genes for the microorganism [14, 73, 74]. Trapped by the BREX system, prophages may serve as ‘lab benches’ for genetic innovation [73]. No homologs of DRI-13^T^’s BREX genes were found with greater than 30% amino acid identity for *pglX* and *brxD* genes and no homologs were found for *brxHI* in subsurface microorganism such as *Candidatus* Desulforudis audaxviator (GCA_000018425.1) or Firmicutes *Desulfotomaculum putei* (GCA_900129195.1). Since the sample pool of deep subsurface microorganisms is small, it would be interesting to see if other obligate subsurface microorganism genomes have similar presence of trapped prophages and putative BREX systems. None of the close relatives to DRI-13^T^ possessed any genes to a BREX system. The presence of this system may explain why DRI-13^T^ possesses these prophage elements while the other genomes lack prophage sequences. In turn, it could be that the reason why the CRISPR spacers did not map to the prophage in DRI-13^T^ is due to the phage being trapped and not replicating because of BREX.

## Conclusion

In this study, we used a hybrid genome assembly of *Thermanaerosceptrum fracticalcis* strain DRI-13^T^ we could increase the detail of metabolism, respiration, DNA repair, ABC transporters, and cellular defense and introduce the first view of the methylome of this subsurface bacterium. Improvements over prior genome assembly create a more contiguous and accurate representation of the genome from which inferences and hypotheses can be generated. The fragmented nature of the previous assembly hindered the discovery of the prophages and BREX system by having parts of these areas on different contigs or missing altogether, demonstrating the importance of contiguous genome sequences. We anticipated to identify unique genes, epigenetic profiles, or environmentally influenced changes in the genome that provide insight into subsurface microbial community lifestyles. The metabolic and DNA repair pathways identified in DRI-13^T^ are conserved as compared to close relative’s genomes from surface or subsurface environments. Among this group of *Peptococcaceae* it appears a focus for syntrophic specialization supplements their survival. The DRI-13^T^ methylated mC and mA profile seem quite different from surface microbes, like laboratory strain *E. coli* K12. Dcm and Dam appear to rely on novel methylation motifs previously not observed. Further methylation investigations need to be performed to confirm these findings. The combination of observed prophages, novel methylation motfis, and BREX defenses, suggests a robust complex mechanism to contain and control subsurface viral attacks. The key to microbial subsurface survival may not rest on genetic diversity, but rather through specific syntrophic niches and methylation strategies.

## Methods

### Culturing and isolation of genomic DNA

High-molecular-weight DNA was extracted from *T. fracticalcis* strain DRI-13^T^ by cultivating the microorganism in one-liter batch cultures grown on 10 mM fumarate. Custom artificial groundwater medium (AGM) was composed of per liter 3.6 g 2-[4-(2-hydroxyethyl) piperazin-1-yl] ethanesulfonic acid (HEPES), 1.5 g Na_2_SO_4_, 0.174 g K_2_PO_4_, 0.14 g Resazurin, 0.4 g MgCl_2_•6H_2_O, 0.5 g KCl, 0.268 g NH_4_Cl, 0.25 g NaHCO_3_, 1 mL ATCC Minimal Vitamins (ATCC, Manassas, VA, United States), and 1 mL ATCC Minimal Minerals. AGM was prepared anaerobically using a modified Hungate technique with 600 mg/L Na_2_S•9H_2_O as a reducing agent. Cells were harvested by centrifugation at 15,000 *x g* for 30 min and the DNA was extracted by cetyltrimethyl ammonium bromide (CTAB) treatment followed by mild homogenization, buffer/chloroform, and finally purified/cleaned with QIAquick PCR (Qiagen) purification columns as previously described [75, 76]. Genomic DNA was assayed by spectrophotometry Nano-drop and then stored at -80 °C until needed.

### Library preparation and MinION sequencing

High quality genomic DNA from *T. fracticalcis* strain DRI-13^T^ was prepared using the procedure outlined in Jain, et al. [18]. With one exception, gDNA was not sheared using a Covaris g-TUBE. For this library preparation a SQK-LSK108 Ligation Sequencing Kit 1D from Oxford Nanopore Technologies (ONT) was used. Approximately 5 μg of high quality gDNA was obtained from 1 L of *T. fracticalcis* strain DRI-13^T^ culture (> 5.0 × 10^7^ cells/mL), of which 1.5 μg was used for library preparation and sequencing on an Oxford Nanopore Technologies MinION sequencer. The MinION sequencing was performed as per manufacturer’s guidelines using a R9.4.1 flow cell (FAH57669). MinION sequencing was operated by the Oxford Nanopore Technologies MinKNOW v19.06.7 software.

### Sequencing preprocessing

Illumina data were acquired as outlined previously [1]. Raw Illumina sequencing reads from initial characterization were quality checked using FastQC [77]. Raw FAST5 files generated from MinION were base-called using Guppy (v 3.3.0) on high accuracy base call model, and summary statistics were created with ToulligQC (version 1.3.0 https://github.com/GenomicParisCentre/toulligQC). The base-called FASTQ files had adapter sequences trimmed using Porechop v0.24 (https://github.com/rrwick/Porechop) then were quality filtered with Filtlong v0.2.0 (https://github.com/rrwick/Filtlong). All programs were run on default settings unless otherwise stated.

### Genome assembly

Since no closed reference for the *T. fracticalcis* genome exists, multiple genome assemblies were conducted consisting of short read only, long read only, and hybrid assemblies. Short read assemblies were performed using SPAdes (v3.13.1) [78]. Long read assemblies were created with Canu (v1.8) [79] and Flye (v2.6-g0d65569) [80], and their respective assemblies were initially polished with base-called FastQ files using Medaka (https://github.com/nanoporetech/medaka), followed by polishing with short reads using Pilon (v1.23) [81] until no changes were detected. Hybrid assemblies were conducted using SPAdes hybrid (v3.13.1), MaSuRCA (v3.34), and Unicycler (v0.4.8-beta) hybrid assembly pipelines [78, 82, 83]. Assembly evaluations were performed using Quast (v5.02), and the final choice of draft genome sequence was made based on contiguity and coverage statistics [84]. Genome assemblies containing ambiguous bases or that possessed fragmented sequences were not considered for downstream processing.

### Genome annotation

Draft genome annotation was performed using JGI annotation pipeline (5.0.3 Gold ID- Ga0415097, taxon ID 2842667859) [16, 17]. PHASTER was used to determine the presence or absence of prophage elements imbedded in the genome [85]. CRISPR-Cas++ (v1.1.2) was used to analyze CRISPR sequences for classification and functionality [86, 87]. Genomic comparisons were performed on genome sequences from *Pelotomaculum propionicicum* (JGI Genome ID: 2889842500) and *Pelotomaculum thermopropionicum* (JGI Genome ID: 640427128), representing closest surface relatives to *T. fracticalcis* [87, 88]. Additionally, genomes of a closely related subsurface bacterium *Desulfotomaculum reducens* (JGI Genome ID: 640069310) and a bacterium isolated from a hydrothermal vent *Thermincola ferriacetica* (JGI Genome ID: 2547132462) were used for comparisons to microorganisms from subsurface influenced environments [89, 90]. The DRI-13^T^ genome is available from JGI 2842667859 and NCBI GCA_000746025.2.

### Methylation analysis

Raw signal FAST5 files from MinIon sequencing were quality filtered using ONT’s Guppy base caller software. Raw reads that passed base calling underwent two independent approaches for detection and mapping of DNA methylation: 1) Denovo signal comparison based analysis using ONT’s production DNA methylation detection suite TOMBO [91], 2) Pre-trained signal comparison-based analysis using Nanopolish [28]. We used 30X as coverage cutoff for our methylation calling on CpG, Dcm, and Dam. For asymmetrical DNA methylation in CHH context we used TOMBO’s de novo detection method and de novo DNA prediction of methylation motifs.

DNA methylation level for each individual context was calculated using the following equation:

$$ \mathbf{Per}\ \mathbf{Context}\ \mathbf{Methylation}\%=\frac{\left(\ \mathbf{Sum}\ \mathbf{of}\ \mathbf{individual}\ \mathbf{Nucleotide}\ \mathbf{Methylation}\ \mathbf{Fraction}\right)}{\left(\mathbf{Total}\ \mathbf{number}\ \mathbf{of}\ \mathbf{Called}\ \mathbf{Methylation}\ \mathbf{Motif}\right)} $$

Methylation signal file was processed into a bigwig file for downstream analysis using Deeptools and Bedtools [92], and plotting of methylation was completed using Deeptools [93] with R package ggplot2 [94].

## Supplementary Information

**Additional file 1: Supplemental Figure S1.** Strain DRI-13^T^ metaplot of all genes for given DNA methylation context. A) mCpG, B) Dcm, C) Dam, D) mCHH (forward), E) mCHH (reverse). All gene methylation profile were normalized (1kbp) including 1kbp upstream and downstream. TSS: Transcription Start Site. TTS: Transcription Termination Site.

**Additional file 2: Supplemental Figure S2.** Methylation profile. A) Enriched 6mA motif G (mA)ACT. B) Enriched 5mC motif C (mC)GG. Red box indicates specific given motif. Violin plots shows fraction of modified nucleotides in scanned Fast5 sequences. Three of each de novo detected motif are shown above the violin plot.

**Additional file 3: Supplemental Figure S3.** Conserved core methyltransferase protein sequence for 6mA and 5mC. A) Alignment of strain DRI-13^T^’s DNA adenine methyltransferase protein sequence versus well studied 6mA methyltransferase in selected prokaryote and eukaryote species. B). Alignment of strain DRI-13^T^’s DNA cytosine methyltransferase protein sequence versus well studied 5mC methyltransferase in selected prokaryote and eukaryote species.

**Additional file 4: Supplemental Figure S4.** A sub cluster of strain DRI-13^T^’s CDS shows enriched mCpG. Heatmapping of all strain DRI-13^T^ CDS. Each row represents one gene. Group binned by K-means clustering. Dashed yellow line represents -35bp from TSS.

**Additional file 5: Supplemental Table S1.** Annotated genes

**Additional file 6: Supplemental Table S2.** Genes cluster 1 of unknown protein families

## Data Availability

DNA methylation analysis pipeline follows Nanopolish and TOMBO documentation which can be found on their respective Github page. Nanopolish: https://github.com/jts/nanopolishTOMBO: https://github.com/nanoporetech/tombo. All other processing scripts can be found on the Hamilton-Brehm-Laboratory GitHub page. Methylation signal data are available in bigwig format upon request to the author. DRI-13^T^ genome is available from JGI 2842667859 and NCBI GCA_000746025.2.

## Abbreviations

ONT: Oxford Nanopore Technology
gDNA: genomic DNA
5mC: 5 methyl cytosine
6 mA: 6-methyl adenine
mCHH: methyl cytosine and H means A, C or T
Dam: DNA adenine methyltransferase
Dcm: DNA cytosine methyltransferase
mCpG: methyl cytosine phosphate guanine
