# Supplementary figures and images for: Hybrid genome de novo assembly with methylome analysis of the anaerobic thermophilic subsurface bacterium Thermanaerosceptrum fracticalcis strain DRI-13T
Source: BMC Genomics. 2021 Mar 23;22:209. doi: 10.1186/s12864-021-07535-z (PMC7988955; doi:10.1186/s12864-021-07535-z)

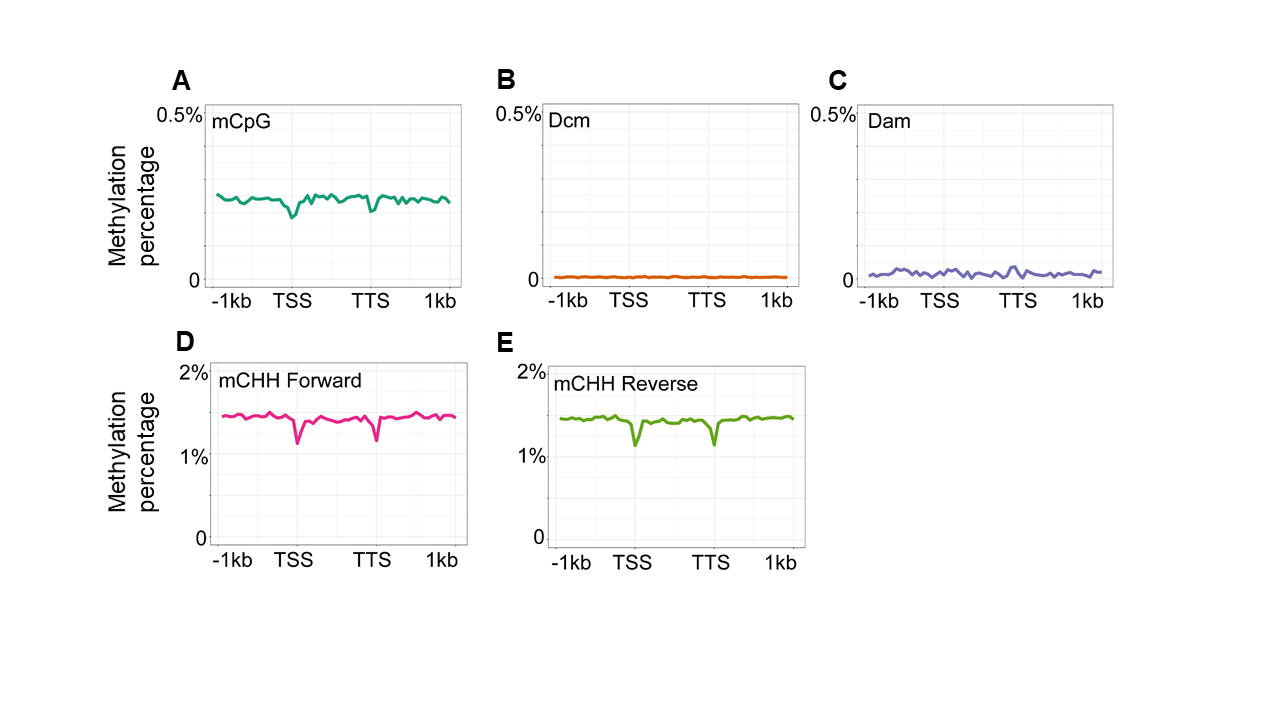

Supplement: Supplementary file 1 — Additional file 1: Supplemental Figure S1. Strain DRI-13T metaplot of all genes for given DNA methylation context. A) mCpG, B) Dcm, C) Dam, D) mCHH (forward), E) mCHH (reverse). All gene methylation profile were normalized (1kbp) including 1kbp upstream and downstream. TSS: Transcription Start Site. TTS: Transcription Termination Site. [file 12864_2021_7535_MOESM1_ESM.tif]

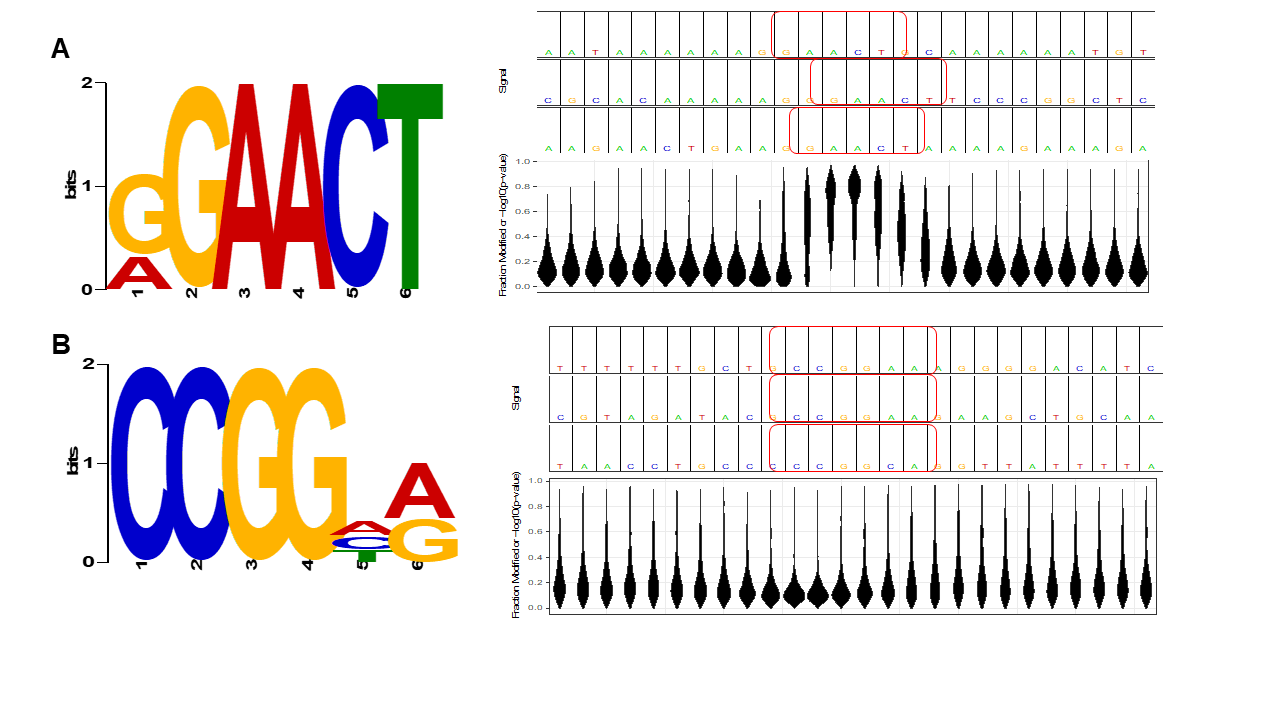

Supplement: Supplementary file 2 — Additional file 2: Supplemental Figure S2. Methylation profile. A) Enriched 6mA motif G (mA)ACT. B) Enriched 5mC motif C (mC)GG. Red box indicates specific given motif. Violin plots shows fraction of modified nucleotides in scanned Fast5 sequences. Three of each de novo detected motif are shown above the violin plot. [file 12864_2021_7535_MOESM2_ESM.tif]

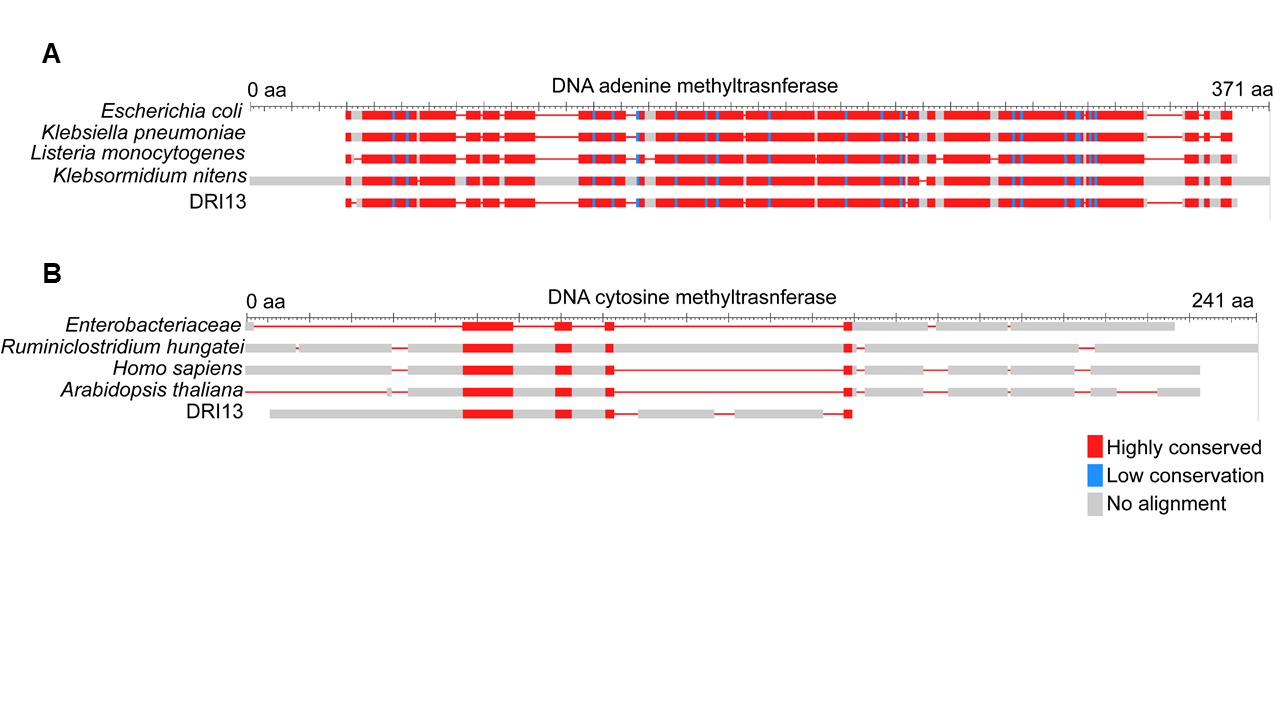

Supplement: Supplementary file 3 — Additional file 3: Supplemental Figure S3. Conserved core methyltransferase protein sequence for 6mA and 5mC. A) Alignment of strain DRI-13T’s DNA adenine methyltransferase protein sequence versus well studied 6mA methyltransferase in selected prokaryote and eukaryote species. B). Alignment of strain DRI-13T’s DNA cytosine methyltransferase protein sequence versus well studied 5mC methyltransferase in selected prokaryote and eukaryote species. [file 12864_2021_7535_MOESM3_ESM.tif]

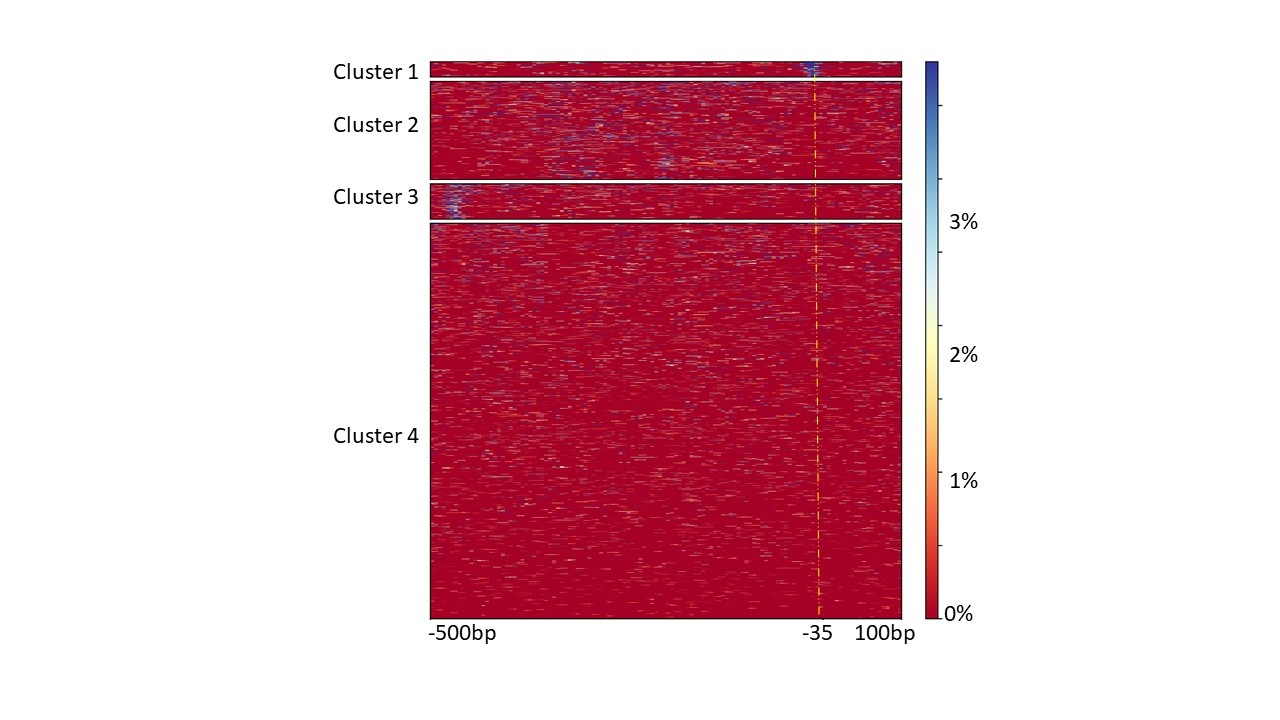

Supplement: Supplementary file 4 — Additional file 4: Supplemental Figure S4. A sub cluster of strain DRI-13T’s CDS shows enriched mCpG. Heatmapping of all strain DRI-13T CDS. Each row represents one gene. Group binned by K-means clustering. Dashed yellow line represents -35bp from TSS. [file 12864_2021_7535_MOESM4_ESM.tif]
